# Effects of Tea Polyphenols and Theaflavins on Three Oral Cariogenic Bacteria

**DOI:** 10.3390/molecules28166034

**Published:** 2023-08-12

**Authors:** Xia Cui, Lei Xu, Kezhen Qi, Hai Lan

**Affiliations:** 1 College of Pharmacy, Dali University, Dali 671000, China; 2 College of Fundamentals and Pharmacy, Yunnan Medical Health College, Anning 650300, China

**Keywords:** tea polyphenols, theaflavins, oral cariogenic bacteria, biofilm, effect of inhibition

## Abstract

In order to investigate the antibacterial mechanism of tea polyphenols and theaflavins against oral cariogenic bacteria, the pH value of the culture medium, the number of bacteria adhering to the smooth glass tube wall, and the electrical conductivity value within 10 h were measured, respectively. The effects of four concentrations of tea polyphenols and theaflavins below the MIC value were studied on acid production, adhesion, and electrical conductivity of oral cariogenic bacteria. The live/dead staining method was used to observe the effects of four concentrations of tea polyphenols and theaflavins below the MIC value on the biofilm formation of oral cariogenic bacteria under a laser scanning confocal microscope. With the increase in concentrations of tea polyphenols and theaflavins, the acid production and adhesion of the cariogenic bacteria gradually decreased, and the conductivity gradually increased. However, the conductivity increase was not significant (*p* < 0.05). Compared with the control group, the 1/2MIC and 1/4MIC tea polyphenols and theaflavins treatments significantly reduced the biomass of the cariogenic biofilm (*p* < 0.05). The confocal laser scanning microscope showed that the integrated optical density of green fluorescence of the cariogenic biofilm gradually decreased with the increase in agent concentration after the action of tea polyphenols and theaflavins.

## 1. Introduction

Dental caries is a common human oral disease with high incidence and comprehensive coverage. Modern studies have demonstrated that human caries are mainly due to oral bacterial infection. Oral bacteria are divided into *Mutansstreptococci*, *Lactobacillus Beijerinck*, and *Actinomyces* [1,2]. Caries is essentially a microbial ecological imbalance disease, and cariogenic bacteria such as *Streptococcus mutans* are all normal oral microbiota and belong to conditional pathogenic bacteria [3,4]. The occurrence of dental caries is mainly due to the imbalance in oral flora. Many cariogenic bacteria synthesize extracellular polysaccharides to attach to the tooth surface to form dental plaque, convert sugars into organic acids, and attach to the enamel surface, decreasing the pH value of the microenvironment of the enamel surface. H^+^ enters the enamel and leads to enamel demineralization and the dissolution of apatite crystals, which destroys the inorganic calcium and phosphorus. Therefore, the most effective way to prevent dental caries is to inhibit the growth of cariogenic bacteria, reduce the formation of organic acids, and prevent enamel demineralization. Domestic and foreign scholars have conducted extensive research on preventing dental caries from aspects of antibiotics, fluoride, and immunology. However, long-term use of chlorhexidine inevitably causes oral erosion and discoloration. Fluoride can lead to dental fluorosis and other toxic side effects with increased fluoride concentration. The method of immunological caries prevention exposes problems in human application.Therefore, the search for a safe anticaries drug that can effectively regulate the balance of oral flora has attracted much attention. Tea, the most popular beverage globally, has been consumed in China for thousands of years [5]. Tea is a natural oral health substance that has been increasingly studied. Many studies have confirmed that tea polyphenols (TP) can inhibit the growth, acid production, adhesion, and accumulation of cariogenic bacteria on the tooth surface and enhance the acid resistance of tooth hard tissues, thereby reducing the occurrence of dental caries [6,7]. Studies have shown that tea pigments formed by oxidative polymerization of TP may have stronger medicinal and healthcare functions than TP, as the main components of tea pigments and theaflavins (TFs) are flavonoids with antioxidant, antibacterial, and anti-inflammatory effects [8]. Bedran et al. found that TFs can significantly inhibit the growth and acid production of major oral pathogens [9]. Relevant mechanism analysis showed that TFs strongly inhibited the glucosyltransferase (GTF) of *Streptococcus mutans*. TFs have a strong affinity with α-salivary amylase. They can inhibit the activity of α-amylase by 50% at low concentrations of 0.6–1.7 μmol/L, thereby interfering with the ability of bacteria to decompose sucrose and starch into glucose and maltose and inhibiting acid production [10,11,12,13].

In recent years, research on preventing dental caries at home and abroad has focused on tea extracts inhibiting the growth of oral cariogenic bacteria. However, there needs to be more understanding of how to inhibit the growth of cariogenic bacteria. In this study, two tea extracts, TP and TFs, were used to further confirm the efficacy of tea as an anticaries agent by inhibiting the production of acid and adhesion and altering the cell membrane permeability of oral cariogenic bacteria.

## 2. Results

### 2.1. Determination of MIC and MBC of Tea Polyphenols and Theaflavins against Cariogenic Bacteria

The results of the inhibition of cariogenic bacteria by tea polyphenols and theaflavins are shown in Table 1. For *Streptococcus mutans*, 95% TP showed the best inhibitory effect with an MIC of 2 mg/mL, followed by 40% TFs with a 4 mg/mL MIC. The bactericidal effect of 40% TFs was similar to that of 95% TP, with an MBC of 16 mg/mL. Compared with 95% TP, 40% TFs (MIC = 2 mg/mL) had the same antibacterial effect on *Streptococcus sobrinus*. The bactericidal effect of 40% and 60% of TFs was similar to 95% of the TP, and the MBC was 8 mg/mL. TP with 95% purity had the best antibacterial effect for *Actinomyces viscosus* with an MIC of 1 mg/mL, followed by 40% TFs with an MIC of 2 mg/mL. TP with 95% purity had the best bactericidal effect, with an MBC of 4 mg/mL, followed by TFs with 20%, 40%, and 60% purity, with an MBC of 32 mg/mL.

### 2.2. Cariogenic Bacteria Acidogenicity after Treatment with Tea Polyphenols and Theaflavins 

**Figure 1:**
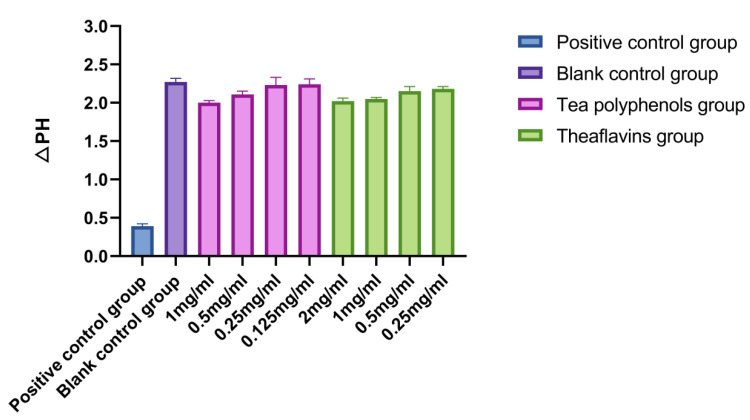
Effects of tea polyphenols and theaflavins on acid production by *S.m*.

**Figure 2:**
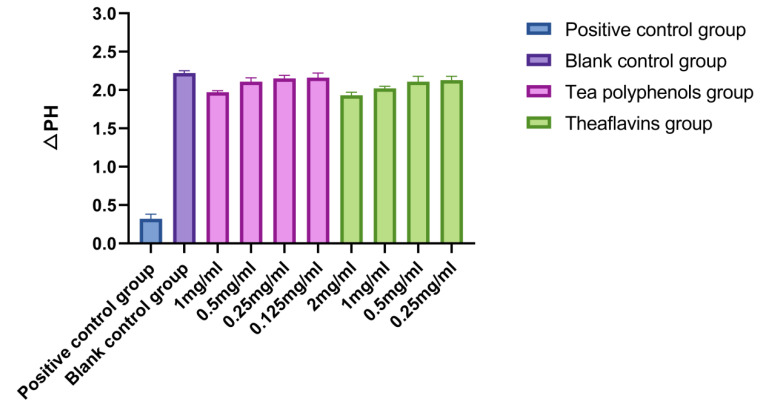
Effects of tea polyphenols and theaflavins on acid production by *S.s*.

**Figure 3:**
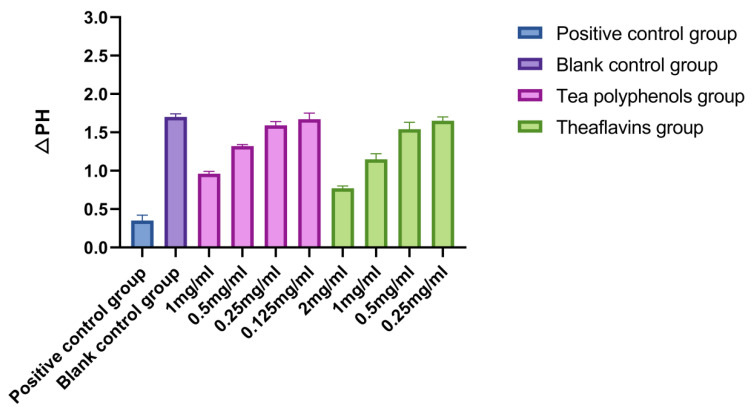
Effects of tea polyphenols and theaflavins on acid production by *A.v*.

The results of the effects of tea polyphenols and theaflavins on acid production of cariogenic bacteria are shown in Figure 1, Figure 2 and Figure 3 and Table S1. For cariogenic bacteria, the acid-producing and acid-resistant ability are as follows: *Streptococcus mutans* > *Streptococcus sobrinus* > *Actinomyces viscosus*. When the pH in the environment decreases to 5.03, *Streptococcus mutans* can still grow. With the increase of the concentration of tea polyphenols and Theaflavin, the inhibition of acid production of cariogenic bacteria also increased. For *Streptococcus mutans*, subinhibitory concentrations of tea polyphenols and theaflavins significantly reduced acid production compared with the control group (*p* < 0.05). For *Streptococcus sobrinus*, the acid production of 1/2MIC tea polyphenol and 1/2MIC and 1/4MIC theaflavins was significantly lower than that of the blank control group (*p* < 0.05). For *Actinomyces viscosus*, the acid production of 1/2MIC and 1/4MIC tea polyphenols and 1/2MIC and 1/4MIC theaflavins was significantly less than that of the blank control group (*p* < 0.05). The acid production of bacteria by four different concentration gradients of the flavanols and tea polyphenols was studied. It was found that as the concentration of the drug increased, the pH value of the experimental environment decreased, and the ΔpH value also decreased. There was an inverse relationship between the acid-producing capacity of bacteria and the agent concentration.

### 2.3. Effects of Tea Polyphenols and Theaflavins on the Adhesion of Cariogenic Bacteria

**Figure 4:**
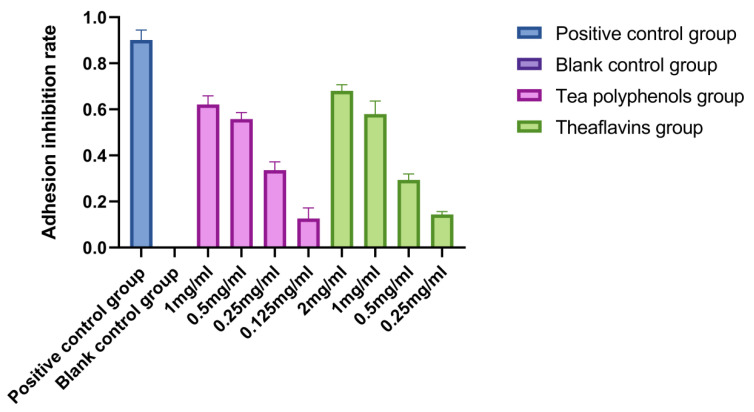
Effects of tea polyphenols and theaflavins on the adhesion of *S.m*.

**Figure 5:**
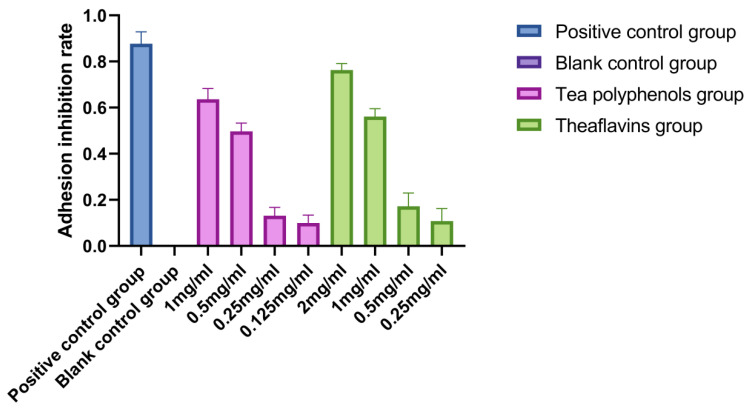
Effects of tea polyphenols and theaflavins on the adhesion of *S.s*.

**Figure 6:**
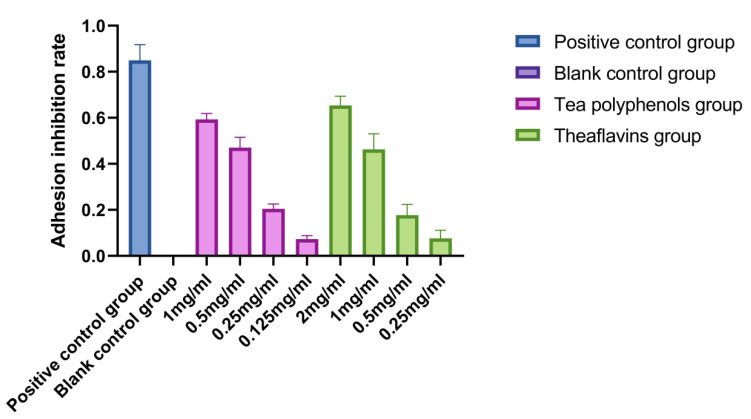
Effects of tea polyphenols and theaflavins on adhesion of *A.v*.

The adhesion of *Streptococcus mutans* is an important prerequisite for the formation of dental caries. *Streptococcus mutans* can firmly adhere to the surface of teeth, use carbohydrates in food to produce organic acids, and significantly decrease the pH of their living environment, resulting in enamel demineralization and the formation of dental caries [14,15,16]. The adhesion of *Streptococcus sobrinus* is similar to that of *Streptococcus mutan*. Both adhere to the tooth surface to form dental plaque and produce acid in the metabolic environment to cause demineralization of tooth tissue, leading to dental caries. The results of the effects of tea polyphenols and theaflavins on the adhesion of cariogenic bacteria are shown in Figure 4, Figure 5 and Figure 6 and Table S2. The adhesion ability of cariogenic bacteria was as follows: *Actinomyces viscosus* > *Streptococcus sobrinus* > *Streptococcus mutans*. With the increase in agent concentration, the adhesion of bacteria on the smooth glass surface gradually decreased, and the adhesion inhibition rate also decreased. However, the inhibition effect was not as good as that of the positive control group. For *Streptococcus mutans*, the adhesion inhibition rates of tea polyphenols and theaflavins in all agent concentration groups were significantly higher than those in the blank control group (*p* < 0.05). For *Streptococcus sobrinus*, except for 1/16MIC tea polyphenols and 1/16MIC theaflavins, the adhesion inhibition rates of the other groups were significantly higher than those of the blank control group (*p* < 0.05). For *Actinomyces viscosus*, except for 1/16MIC of tea polyphenols and 1/8MIC and 1/16MIC of theaflavins, the adhesion inhibition rates of the other groups were significantly higher than those of the blank control group (*p* < 0.05).

### 2.4. Effects of Tea Polyphenols and Theaflavins on the Conductivity of Cariogenic Bacteria Culture Medium

**Figure 7:**
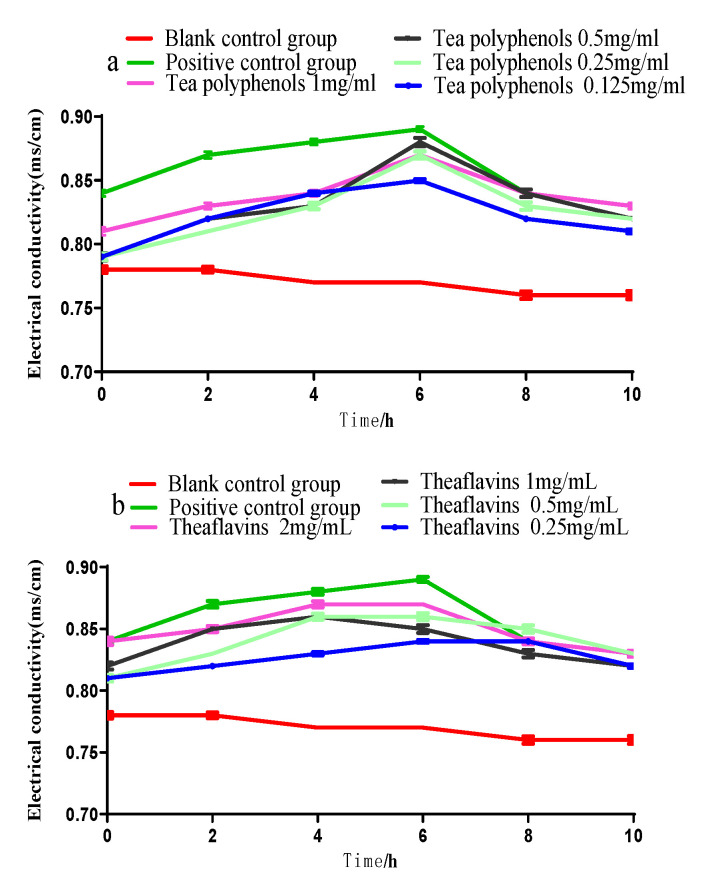
Conductivity of *S.m* culture medium after treatment with tea polyphenols (**a**) and theaflavins (**b**).

**Figure 8:**
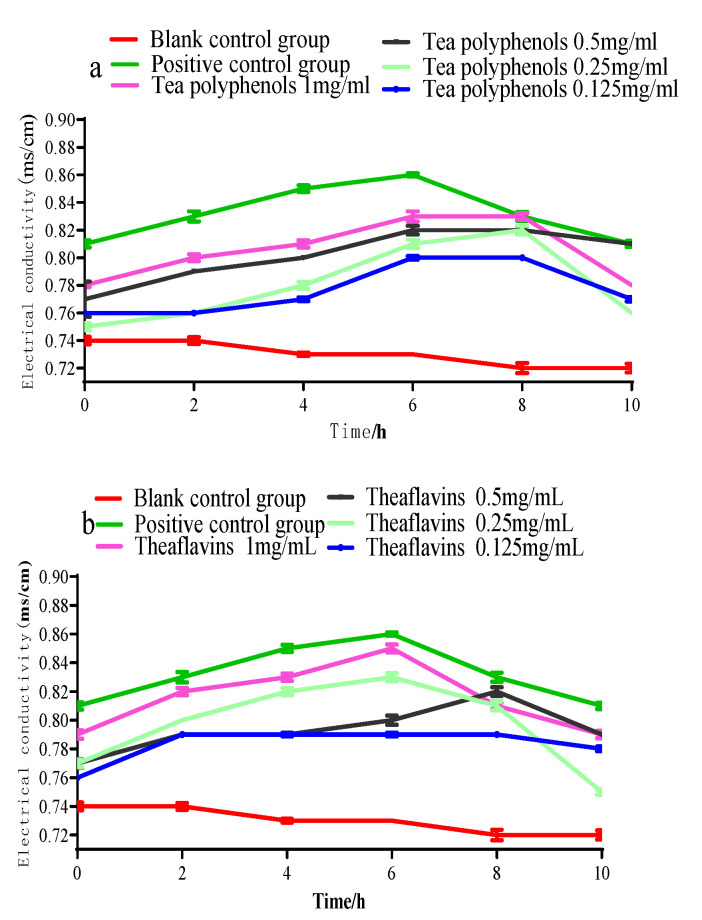
Effects of tea polyphenols (**a**) and theaflavins (**b**) on the conductivity of the culture medium of *S.s*.

**Figure 9:**
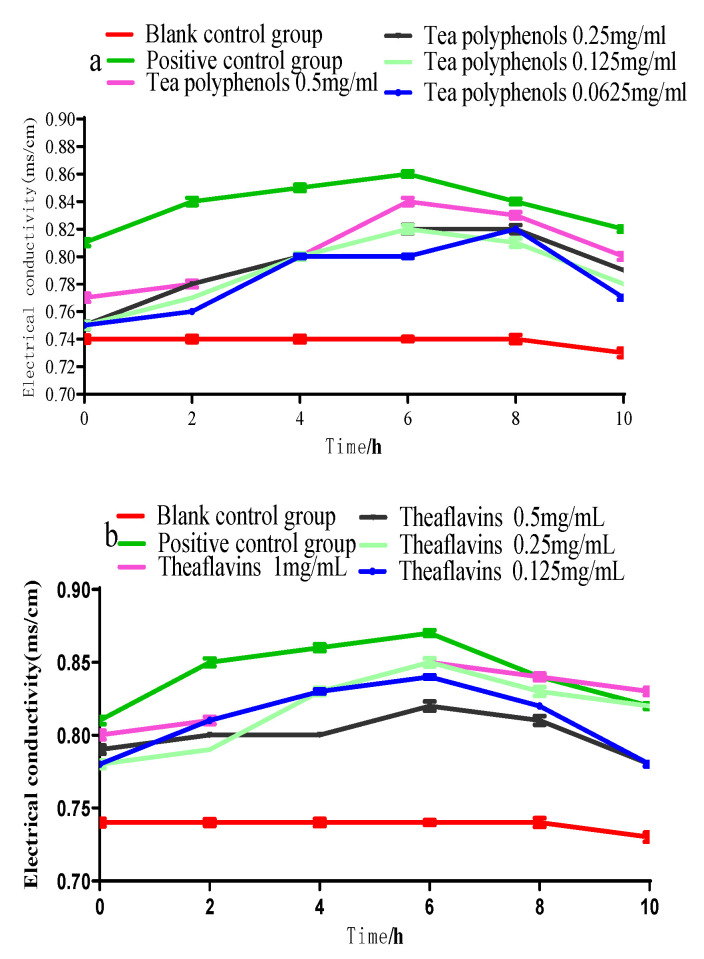
Effects of tea polyphenols (**a**) and theaflavins (**b**) on the conductivity of the culture medium of *A.v*.

The cell membrane is the protective barrier of bacteria. When it is destroyed, the protective barrier of bacteria will be broken, and the internal electrolytes will be released into the culture medium to increase conductivity. The bacteria will be damaged or even killed. The effects of tea polyphenols and theaflavins on the conductivity of the culture medium of cariogenic bacteria are shown in Figure 7, Figure 8 and Figure 9. The conductivity of the culture medium was higher than that of the blank control group after adding the experimental agent solution, which may be due to the charge of some components of tea polyphenols and theaflavins, leading to the increase in the conductivity of the culture medium. As the processing time prolonged, the conductivity of each experimental group gradually increased, but the increase was not significantly (*p* > 0.05). Therefore, it was inferred that polyphenols and theaflavins caused little damage to the cell membrane of erotogenic bacteria, and their inhibition of erotogenic bacteria did not play a role in damaging the cell membrane.

### 2.5. Effects of Tea Polyphenols and Theaflavins on Cariogenic Biofilm Biomass

**Figure 10:**
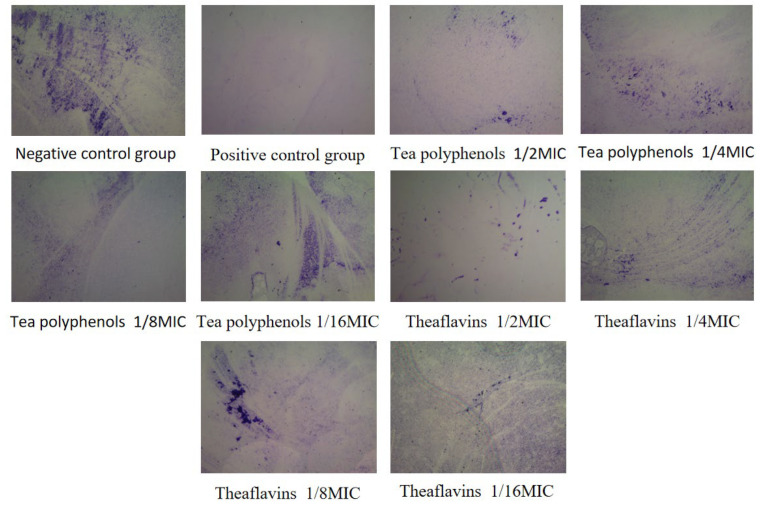
Typical crystal violet images of *S.m* biofilms treated with tea polyphenols and Theaflavin.

**Figure 11:**
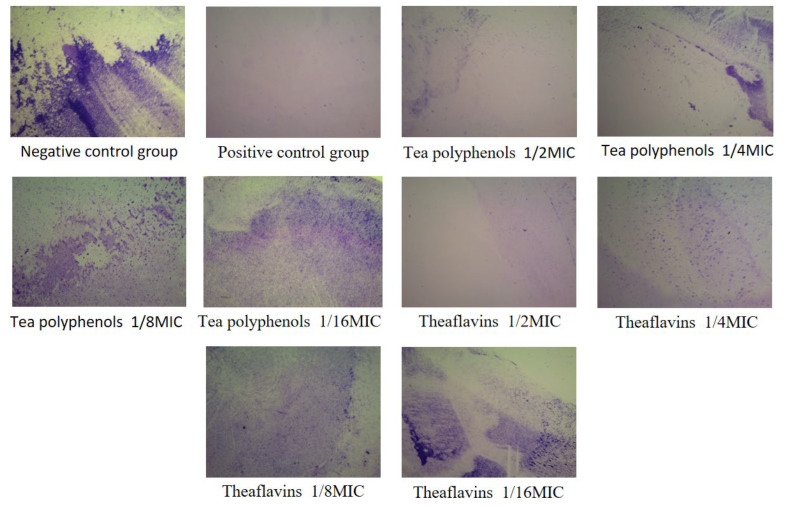
Typical crystal violet images of tea polyphenols and theaflavins against *S.s* biofilms.

**Figure 12:**
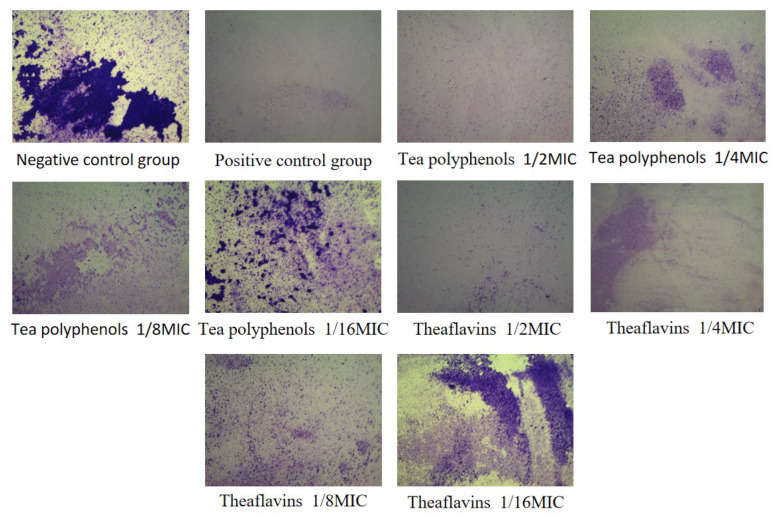
Typical crystal violet images of tea polyphenols and theaflavins against *A.v* biofilms.

**Figure 13:**
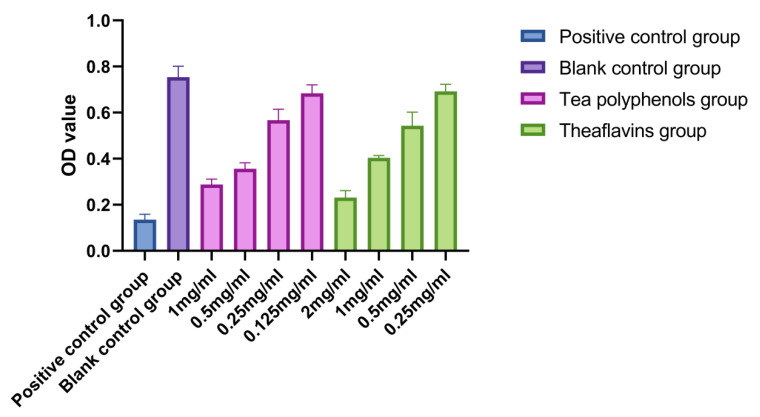
Effect of tea polyphenols and theaflavins on biofilm biomass of *S.m*.

**Figure 14:**
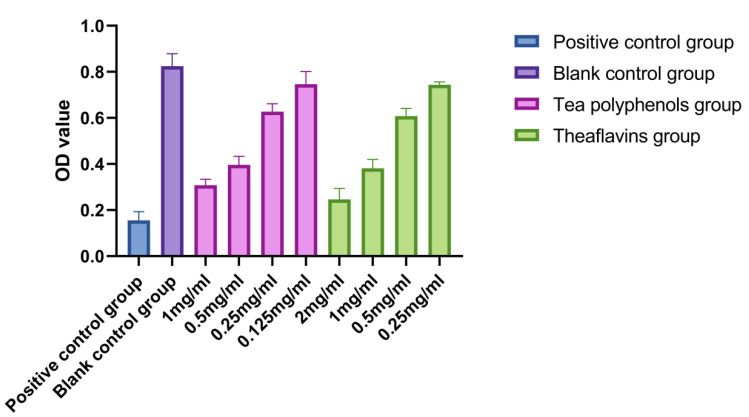
Effects of tea polyphenols and theaflavins on biofilm biomass of *S.s*.

**Figure 15:**
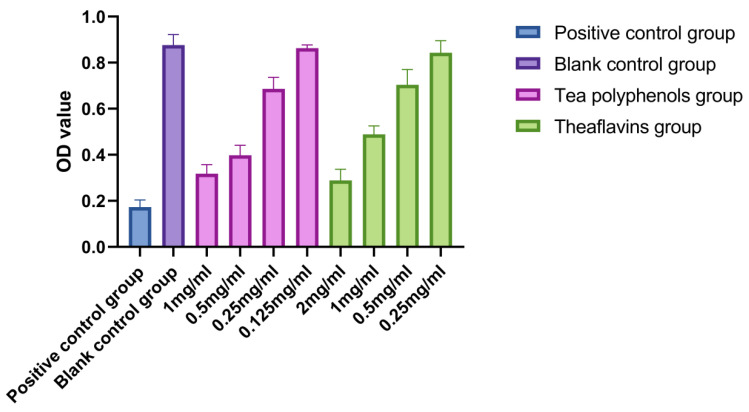
Effects of tea polyphenols and theaflavins on biofilm biomass of *A.v*.

Crystal violet is a basic dye that can stain DNA, proteins, and fat. The staining shows that the nucleus is blue and the cytoplasm is pink, thus staining the nucleus. The results of the effects of tea polyphenols and theaflavins on the biofilm biomass of cariogenic bacteria are shown in Figure 10, Figure 11, Figure 12, Figure 13, Figure 14 and Figure 15 and Table S3. After 48 h of culture, the blank control group and each experimental group formed a layer of gray-white film on the cell slide. The blank control group had the largest area of film, and the other experimental groups had scattered biofilm, which was scattered on the slide. After crystal violet staining, the blank control group had a darker crystal violet color and a larger area. Crystal violet staining was light or showed almost no color on the slides of the other experimental groups, and the area was small. As the concentrations of tea polyphenols and theaflavins decreased, the OD value increased, indicating that there was an inverse relationship between the concentration of tea polyphenols and the biomass of cariogenic bacteria biofilm. Compared with the blank control group, the biomass of cariogenic bacteria biofilm was significantly decreased after treatment with 1/2MIC and 1/4MIC tea polyphenols and 1/2MIC and 1/4MIC theaflavins (*p* < 0.05).

### 2.6. Effects of Tea Polyphenols and Theaflavins on the Biofilm Activity of Cariogenic Bacteria

**Figure 16:**
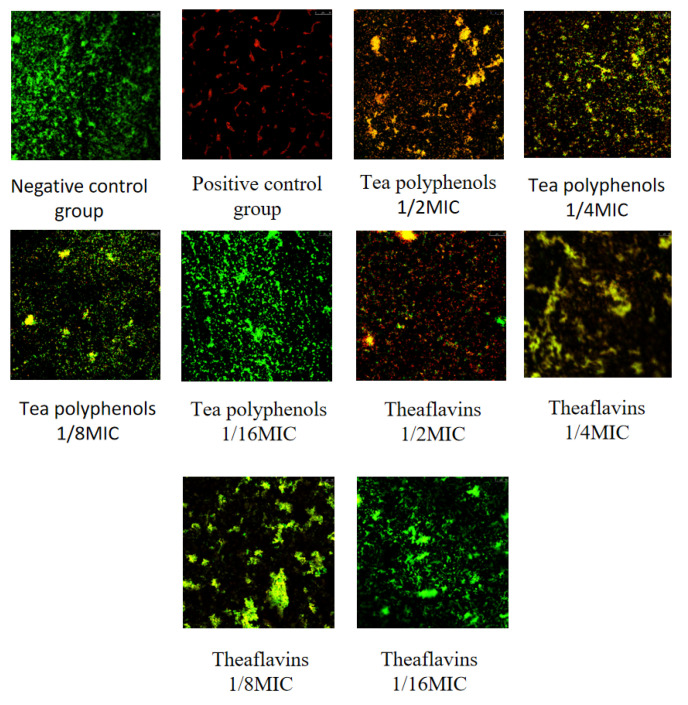
Typical live/dead staining confocal images of tea polyphenols and theaflavins on *S.m* biofilms.

**Figure 17:**
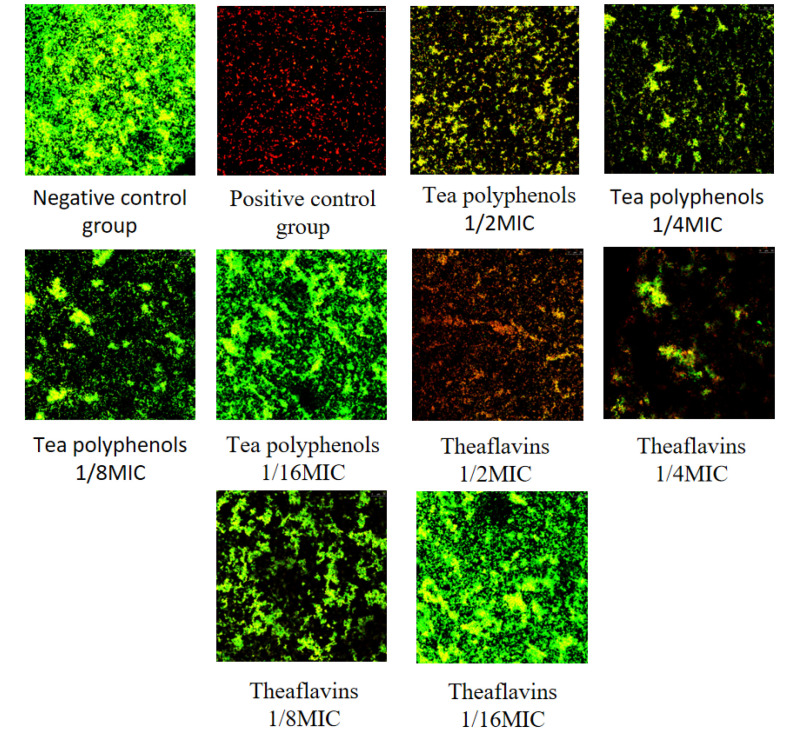
Typical live/dead staining confocal images of tea polyphenols and theaflavins on *S.s* biofilms.

**Figure 18:**
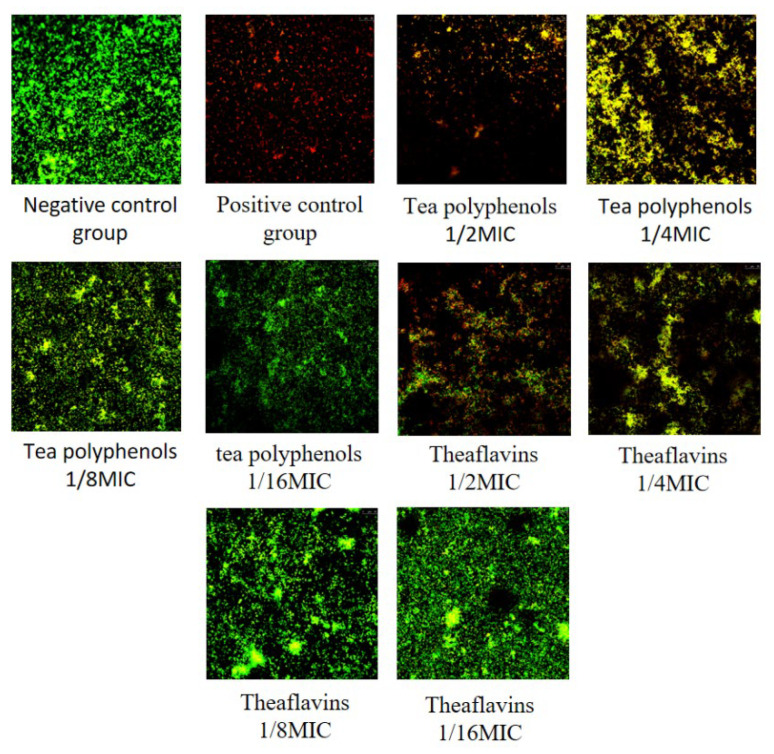
Typical live/dead staining confocal images of tea polyphenols and theaflavins on *A.v* biofilms.

**Figure 19:**
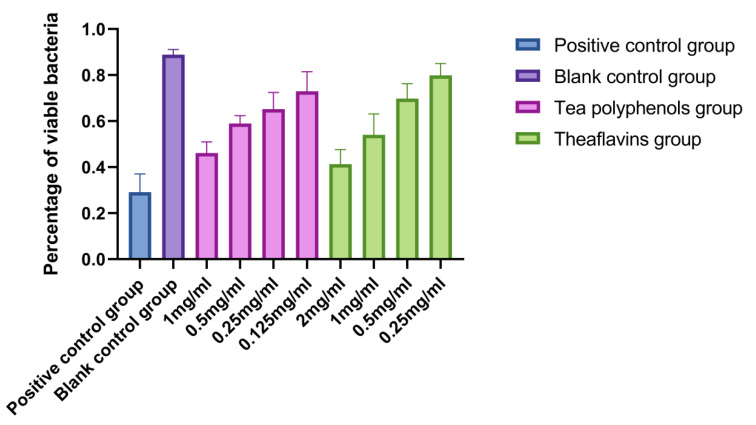
Effect of tea polyphenols and theaflavins on the percentage of viable bacteria in *S.m* biofilms.

**Figure 20:**
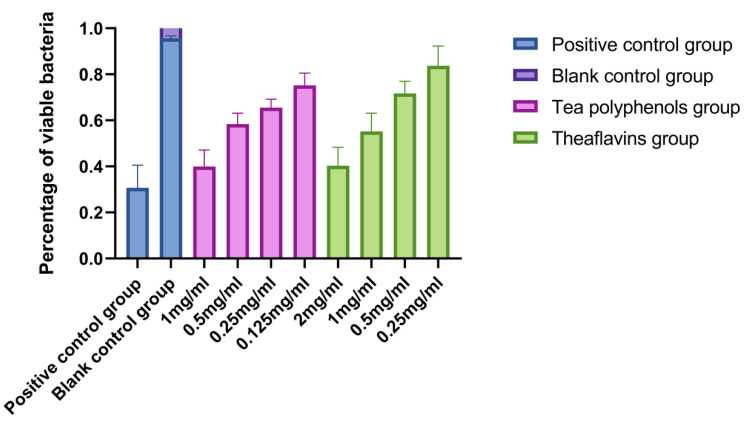
Effect of tea polyphenols and theaflavins on the percentage of viable bacteria in *S.s* biofilms.

**Figure 21:**
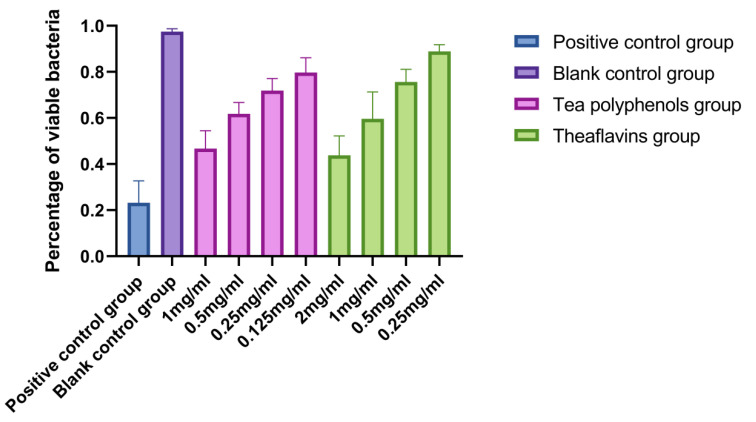
Effect of tea polyphenols and theaflavins on the percentage of viable bacteria in *A.v* biofilms.

The results of the effects of tea polyphenols and theaflavins on the biofilm activity of cariogenic bacteria are shown in Figure 16, Figure 17, Figure 18, Figure 19, Figure 20 and Figure 21 and Table S4. CLSM observed that the integrated optical density of green fluorescence of different tea polyphenols and theaflavins on cariogenic biofilm gradually decreased with the increase in agent concentration, suggesting that various agent concentrations of tea polyphenols and theaflavins affected the morphological structure of biofilm. After staining with LIVE/DEAD^®^BacLight™ bacterial viability kit, the blank control group showed a green patch under the laser scanning confocal microscope with complete biofilm structure, while the positive control group showed a red patch under the microscope with a small number of bacteria, and most of them were dead bacteria. Red–green fluorescence quantitative analysis showed that tea polyphenols at 1/2MIC and 1/4MIC and theaflavins at 1/2MIC could significantly affect the cariogenic biofilm activity compared with the blank control group (*p* < 0.05), indicating that tea polyphenols at 1/2MIC and 1/4MIC and theaflavins at 1/2MIC could affect the cariogenic biofilm activity.

## 3. Methods

### 3.1. Materials

#### 3.1.1. Bacteria and Culture Medium

Strains tested: *Streptococcus mutans* (*S.m* ATCC25175), *Streptococcus sobrinus (S.s* ATCC33478), and *Actinomyces viscosus* (*A.v* ATCC27044) were bought in microbial strains from Guangdong Province Preservation center.

Brain heart infusion (BHI): Guangdong Huankai Microbial Technology Co., LTD.

AgarPower: Solarbio^®^.

#### 3.1.2. Samples

Theaflavins sample: solid orange powder, total theaflavin (including four main components: TF1, TF2A, TF2B, and TF3) content was 20% (batch number: TF20-19092401H), 40% (batch number: TF40-19092401H), 60% (batch number: TF40-19092401H), and 80% (batch number: TF80-19092401H), purchased from Jiangsu Dehe Biotechnology Co., LTD. (tea collected from Wuyuan, Jiangxi Province, China).

Tea polyphenols sample (batch number: TP95-19092401H): light-yellow solid powder with 95% catechin content (studies have shown that tea polyphenols with 95% catechin content have an excellent inhibitory effect on *Streptococcus mutants*, *Streptococcus sobrinus*, and *Actinomyces viscosus*), purchased from Jiangsu Dehe Biological Technology Co., LTD. (tea collected from Wuyuan, Jiangxi Province) The four experimental samples are respectively referred to as theaflavins with purity of 20%, 40%, 60% and 80% and tea polyphenols with purity of 95%. 

#### 3.1.3. Instruments and Reagents

Enzyme-labeled instrument: US Baiteng, model: BIO-Tek Synergy HT; pH meter: Shanghai Yidian Scientific Instrument Co., Ltd., Shanghai, China, model: pHS-3C; Leici DDS-11A digital conductivity meter: Shanghai Leici Chuangyi Instrument Co., Ltd., Shanghai, China, model: DDS-11A; stereoscopic microscope: Chongqing Optical instrument Factory, model: XTL-3C; confocal laser scanning microscope: Leica Microsystems Trading Co., Ltd., model: ALEICA TCS SP8; LIVE/DEAD^®^BacLight™ Bacterial Viability Kit: Kits L7012, Molecular Probes, Eugene, OR, USA.

McFarland turbidimetric tube: Beijing Solaibao Technology Co., Ltd.; chlorhexidine: Shanghai McLean Biochemical Technology Co., Ltd., analytical pure.

### 3.2. Preparation of Bacterial Solution

After 48 h of recovery, the strains were incubated with BHI solid medium for 18 h at 37 °C, under anaerobic conditions of 80% N_2_, and 20% CO_2_. After biochemical identification and smear examination, a single colony was selected and cultured in BHI liquid medium under the same conditions for 18 h, and the concentration of bacterial suspension was adjusted to 1 × 10^8^ CFU/mL by McFarland turbidimetric tube.

### 3.3. Preparation of Positive Control

Chlorhexidine was precisely weighed, prepared to a 0.05% concentration solution, and placed in a 4 °C refrigerator until use [17].

### 3.4. Preparation of Agent-Containing Solution

The tea polyphenols and theaflavins were, respectively, dissolved in BHI liquid medium by twofold dilution method so that the test agent concentration was 0.25 mg/mL, 0.5 mg/mL, 1 mg/mL, 2 mg/mL, 4 mg/mL, 8 mg/mL, 16 mg/mL, 32 mg/mL, 64 mg/mL, and 128 mg/mL, respectively, and refrigerated at 4 °C until use.

### 3.5. MIC and MBC Determination of Tea Polyphenols and Theaflavins against Cariogenic Bacteria

The agent-containing culture solution prepared in an amount of 2 mL was put into a test tube, and 20 µL of the prepared bacterial solution was added to the test tube. The agent was placed at 37 °C, 80% N_2_, and 20% CO_2_ under anaerobic conditions for 24 h. The minimum inhibitory concentration without bacterial growth observed by the naked eye was taken as the MIC value. Each tube with a concentration greater than the MIC was selected, and 20 µL of each tube was coated on BHI solid medium and incubated at 37 °C, 80% N_2_, and 20% CO_2_ under anaerobic conditions for 48 h. The minimum bactericidal concentration with less than 5 to 6 colonies was observed as the MBC. The experiment was repeated 3 times.

### 3.6. Acid Production of Cariogenic Bacteria treated by Tea Polyphenols and Theaflavins

According to the MIC results of theaflavins and tea polyphenols against the experimental strains, four concentration gradients below the MIC value were selected to prepare the BHI liquid medium, which was refrigerated at 4 °C for later use.

After inoculation with 2.1 prepared bacteria at the ratio of 1:10 (*v*/*v*) between bacterial suspension and BHI agent-containing liquid medium, the bacteria were incubated at 37 °C, 80% N_2_, and 20% CO_2_ under 2 anaerobic conditions for 48 h, and centrifuged at 3000 r/min for 10 min. The pH value of the culture supernatant was measured by a pH meter, and the change in pH (ΔpH) was calculated. A negative control group and a positive control group were set up. The negative control group is a culture medium without agents, and the positive control group is a culture medium containing 0.05% chlorhexidine. Each group had three parallel tubes, and the experiment was repeated three times. Here, ΔpH = pH_(initial)_ − pH_(end)_.

### 3.7. Determination of Adhesion of Cariogenic Bacteria by Tea Polyphenols and Theaflavins

According to the MIC results of theaflavins and tea polyphenols against the experimental strains, four concentration gradients below the MIC value were selected to prepare the BHI liquid medium, which was refrigerated at 4 °C for later use. The bacteria were inoculated at the ratio of 1:10 (*v*/*v*) between bacterial suspension and BHI agent-containing liquid medium. Each test tube was placed 30° above the ground and incubated for 48 h at 37 °C, 80% N_2_, and 20% CO_2_ under anaerobic conditions. The solution in the test tube was removed, and the bacteria adhering to the tube wall were washed with phosphate buffer, 3 mL each time, 3 times, and centrifuged at 4000 r/min for 15 min to collect the bacterial cell precipitate. The bacterial cell precipitate was dispersed with 1 mL of phosphate buffer, and 200 µL was placed in a 96-well plate. The absorbance value was measured at a wavelength of 560 nm. A negative control group and a positive control group were set up. The negative control group is a culture medium without agents, and the positive control group is a culture medium containing 0.05% chlorhexidine. Each group had three parallel tubes, the experiment was repeated three times, and the adhesion inhibition rate was calculated. Adhesion inhibition rate = [1 − experimental group A560 nm/blank control group A560 nm] × 100%.

### 3.8. Determination of the Electrical Conductivity of the Culture Medium of the Cariogenic Bacteria by Tea Polyphenols and Theaflavins

According to the MIC results of theaflavins and tea polyphenols against the experimental strains, four concentration gradients below the MIC value were selected to prepare the BHI liquid medium. The medium was refrigerated at 4 °C for later use.

The bacteria were inoculated at a ratio of 1:10 (*v*/*v*) between bacterial suspension and BHI liquid medium and then incubated at 37 °C, 80% N_2_, and 20% CO_2_ under anaerobic conditions with shaking. The supernatant was diluted 20 times, and the conductivity was measured by a conductivity meter after centrifugation at 4000 r/min for 15 min. The culture medium was cultured for 0, 2, 4, 6, 8, and 10 h. A negative control group and a positive control group were set up in the experiment. The negative control group is a culture medium without agents, and the positive control group is a culture medium containing 0.05% chlorhexidine. Each group had three parallel tubes, and the experiment was repeated three times.

### 3.9. Determination of Cariogenic Biofilm Biomass by Tea Polyphenols and Theaflavins

According to the MIC results of theaflavins and tea polyphenols against the experimental strains, four concentration gradients below the MIC value were selected to prepare a BHI liquid medium, which was filtered by a microporous filter membrane (0.22 μm) and then refrigerated in the refrigerator at 4 °C for later use. Cell slides with a diameter of 25 mm were placed into 6-well cell culture dishes. Amounts of 500 µL of bacterial suspension and 1500 µL of BHI liquid medium were added to each well and cultured at 37 °C, 80% N_2_, and 20% CO_2_ under anaerobic conditions for 48 h to form biofilms. The slides were washed 3 times with deionized water. The biofilms were fixed in formaldehyde for 15 min and stained with 1% crystal violet for 5 min. The biofilms were washed 3 times again with deionized water and captured by stereomicroscope. Then, 200 µL of ethanol solution was transferred to a 96-well plate, and the absorbance at 595 nm was measured using a microplate reader. Each group had three parallel tubes, and the experiment was repeated three times.

### 3.10. Determination of the Biofilm Activity of Tea Polyphenols and Theaflavins against Cariogenic Bacteria

According to the MIC results of theaflavins and tea polyphenols against the experimental strains, four concentration gradients below the MIC value were selected to prepare a BHI liquid medium, filtered through a microporous filter membrane (0.22 μm), and then refrigerated in a refrigerator at 4 °C for later use.

Cell slides with a diameter of 25 mm were placed into 6-well cell culture dishes, and 500 µL bacterial suspension and 1500 µL BHI liquid medium were added to each well. Biofilm was cultured at 37 °C, 80% N_2_, and 20% CO_2_ under anaerobic conditions for 48 h. The medium in the wells was removed, and the wells and slides were washed 3 times with PBS buffer. After 24 h of incubation, the slides were removed and washed thrice with deionized water. The biofilms were fixed in formaldehyde for 15 min. Then, the bacteria viability was measured with the LIVE/DEAD^®^BacLight™ Bacterial Viability Kit moland stained in the dark for 15 min, and the slides were washed 3 times with deionized water. The samples were placed under a laser scanning confocal microscope to capture the biofilm images, and each biofilm was scanned at 5 randomly selected locations. Image-Pro Plus 6.0 software analyzed the amount of live and dead bacteria based on the integrated optical density (IOD), detailed previously by Cui et al. and Zeng et al. [18,19]. The percentage of viable bacteria in the biofilm was calculated. Each group had three parallel tubes, and the experiment was repeated three times. Percentage of viable bacteria = [integrated optical density of green light/(integrated optical density of green light + red light)] × 100%.

## 4. Discussion

*Streptococcus mutans*, *Streptococcus sobrinus*, and *Actinomyces viscosus,* used in this experiment, are the main cariogenic bacteria in the oral cavity. They can ferment various carbohydrates to produce acids, reduce plaque pH below 5, and synthesize intracellular and extracellular polysaccharides using sucrose as a substrate [20,21]. They also have a high affinity for bacterial biofilms, which determines their important role in the occurrence of dental caries [22]. Their growth is closely related to the occurrence of dental caries. This experiment proves that tea polyphenols and theaflavins can reduce the pathogenicity of oral bacteria by inhibiting bacterial growth, acid production, and adhesion, and determines the ability of theaflavins to inhibit oral bacteria biofilm for the first time.

Four different concentration gradients of theaflavins and tea polyphenols were selected in order to study the acid production of bacteria. It was found that with the increase in drug concentration, the pH value of the experimental environment decreased, and the ΔpH value decreased [23]. The acid production capacity of bacteria was inversely proportional to the drug concentration [24]. Some studies have indicated that the acid production ability of *Streptococcus mutans* is mainly related to lactate dehydrogenase and the ATP proton transporter [25]. The mechanism of tea polyphenols and theaflavins inhibiting the acid production of *Streptococcus mutans* in this experiment may be that the liquid itself inhibits the growth of bacteria, directly reduces the amount of acid produced, inhibits GTF activity, and reduces bacterial metabolism [26]. In addition, since sugars in food are substrates of bacterial metabolism, there may be enzymes that inhibit salivary amylase and acid production, but these inferences need to be proven by experiments.

There are two adhesion mechanisms: one is sucrose-independent adhesion, and the other is sucrose-dependent adhesion [27]. Sugar-independent adhesion refers to the adhesion of SM to tooth surfaces in the absence of sucrose. Sugar-dependent adhesion refers to the use of sucrose by Sm to produce water-insoluble glucans, which promote the adhesion and aggregation of Sm on tooth surfaces [28,29,30]. The adhesion of *Actinomyces viscosus* to the tooth surface is related to the fimbriae of *Actinomyces viscosus*. There are two types of *Actinomyces viscosus* fimbriae, type I and type II, which are both involved in bacterial adhesion. *Actinomyces viscosus* fimbriae bind to salivary protein receptors in the mouth [31,32]. The bacteria can bind to the receptor on the acquired membrane through the lectin, adhere to the tooth surface, and grow. *Actinomyces viscosus* have strong surface hydrophobicity, promoting their adhesion to tooth surfaces.

Dental plaque is a naturally existing ecosystem composed of various microbial species, and the communities are in dynamic equilibrium under healthy conditions [33]. Oral diseases happen when changes of environmental factors trigger a deleterious shift in the microbial balance to a pathogenic-associated composition [34,35,36]. From the ecological view, since it is impossible to eliminate all bacteria, maintaining microbial homeostasis is important for preventing and treating dental caries. This study provides prima facie evidence that the biomass and activity of cariogenic bacteria biofilm were significantly reduced after treatment with tea polyphenols and Theaflavin (*p* < 0.05). CLSM observed that as the concentration of tea polyphenols and theaflavins decreased, the biomass of cariogenic bacteria biofilm increased, the integrated optical density of green fluorescence of cariogenic bacteria biofilm increased, and the activity of biofilm increased.

## 5. Conclusions

Tea polyphenols and theaflavins have antibacterial and bactericidal effects on cariogenic bacteria, among which 95% tea polyphenols and 40% theaflavins have better effects. With the increase of the concentration of tea polyphenols and Theaflavin, the inhibitory effect on cariogenic bacteria gradually increases.With the increase in tea polyphenols and theaflavins, the cariogenic bacteria’s acid production and adhesion decreased gradually, and conductivity increased gradually. However, the conductivity increase was not significant (*p* < 0.05). Effects of tea polyphenols and theaflavins on the cariogenic biofilm were as follows: with the decrease in the concentrations of tea polyphenols and theaflavins, the biomass of the cariogenic biofilm increased, the integrated optical density of green fluorescence of the cariogenic biofilm increased, and the biofilm activity increased. Thus, tea polyphenols and theaflavins can be used as a potential anticaries drug.

## Figures and Tables

**Table 1:** Determination of MIC and MBC of cariogenic bacteria by tea polyphenols and theaflavins.

Groups	MIC (mg/mL)			MBC (mg/mL)		
	*S.m*	*S.s*	*A.v*	*S.m*	*S.s*	*A.v*
95% tea polyphenols	2	2	1	16	8	4
20% theaflavins	8	4	4	32	16	32
40% theaflavins	4	2	2	16	8	32
60% theaflavins	8	4	4	32	8	32
80% theaflavins	16	4	16	32	16	64

## Data Availability

The data presented in this study are available on request from the corresponding authors.

## Supplementary Materials

The following are available online at https://www.mdpi.com/article/10.3390/molecules28166034/s1, Table S1: Effects of tea polyphenols and theaflavins on acid production by cariogenic bacteria; Table S2: Effects of tea polyphenols and theaflavins on adhesion inhibition rates of cariogenic bacteria; Table S3: Effects of tea polyphenols and theaflavins on biofilm biomass of cariogenic bacteria; Table S4: Effects of tea polyphenols and theaflavins on the percentage of viable bacteria in the cariogenic biofilm.

## Sample Availability

Samples of the compounds are available from the authors.

## Abbreviations

GTF	glucosyltransferase
TP	tea polyphenols
TFs	theaflavins
*S.m*	*Streptococcus mutans*
*S.s*	*Streptococcus sobrinus*
*A.v*	*Actinomyces viscosus*
BHI	Brain heart infusion
