# Supplementary material for: Effects of Tea Polyphenols and Theaflavins on Three Oral Cariogenic Bacteria
Source: Molecules. 2023 Aug 12;28(16):6034. doi: 10.3390/molecules28166034 (PMC10458778; doi:10.3390/molecules28166034)
Supplement: Supplementary file 1 [file molecules-28-06034-s001.zip › molecules-2511520-supplementary.pdf]

Supporting Information

Effects of Tea Polyphenols and Theaflavins on Three Oral Cariogenic Bacteria

Xia Cui [1,2], Lei Xu [1], Kezhen Qi [1,*] and Hai Lan[1,*]

[1] College of Pharmacy, Dali University, Dali 671000, China
[2] College of Fundamentals and Pharmacy, Yunnan Medical Health College, Anning 650300, China
* Correspondence: qkzh2003@aliyun.com (K.Q.); lanhai8696@126.com (H.L.)

Table S1. Effects of tea polyphenols and theaflavins on acid production by cariogenic bacteria.

		△ pH		
		S.m	*S.s*	*A.v*
Positive control group		0.39±0.03	0.32±0.06	0.35±0.07
Blank control group		2.27±0.05	2.22±0.03	1.70±0.04
Tea polyphenols	1/2MIC	2.00±0.03$^{*\triangle}$	1.97±0.02$^{*\triangle}$	0.96±0.03$^{*\triangle}$
	1/4MIC	2.11±0.04$^{\triangle}$	2.11±0.05$^{\triangle}$	1.32±0.02$^{*\triangle}$
	1/8MIC	2.23±0.10$^{\triangle}$	2.15±0.04$^{\triangle}$	1.59±0.05$^{\triangle}$
	1/16MIC	2.24±0.07$^{\triangle}$	2.16±0.06$^{\triangle}$	1.67±0.08$^{\triangle}$
Theaflavins	1/2MIC	2.02±0.04$^{*\triangle}$	1.93±0.04$^{*\triangle}$	0.77±0.03$^{*\triangle}$
	1/4MIC	2.05±0.02$^{\triangle}$	2.02±0.03$^{*\triangle}$	1.15±0.07$^{*\triangle}$
	1/8MIC	2.15±0.06$^{\triangle}$	2.11±0.07$^{\triangle}$	1.54±0.09$^{\triangle}$
	1/16MIC	2.18±0.03$^{\triangle}$	2.13±0.05$^{\triangle}$	1.65±0.05$^{\triangle}$

Note: * denotes P<0.05 compared with blank control group, $^{\triangle}$ denotes P<0.05 compared with positive control group.

Table S2. Effects of tea polyphenols and theaflavins on adhesion inhibition rates of cariogenic bacteria.

		Adhesion inhibition rate		
		S.m	*S.s*	*A.v*
Positive control group		0.9014±0.0436	0.8770±0.0521	0.8500±0.0679
Blank control group		0.0000±0.0000	0.0000±0.0000	0.0000±0.0000
Tea polyphenols	1/2MIC	0.6210±0.0374$^{*\triangle}$	0.6365±0.0461$^{*\triangle}$	0.5933±0.0258$^{*\triangle}$
	1/4MIC	0.5580±0.0286$^{*\triangle}$	0.4969±0.0364$^{*\triangle}$	0.4702±0.0455$^{*\triangle}$
	1/8MIC	0.3364±0.0354$^{*\triangle}$	0.1314±0.0353$^{*\triangle}$	0.2049±0.0202$^{*\triangle}$
	1/16MIC	0.1266±0.0455$^{*\triangle}$	0.0992±0.0347$^{\triangle}$	0.0733±0.0146$^{\triangle}$
Theaflavins	1/2MIC	0.6804±0.0265$^{*\triangle}$	0.7632±0.0280$^{*\triangle}$	0.6534±0.0400$^{*\triangle}$
	1/4MIC	0.5792±0.0572$^{*\triangle}$	0.5615±0.0343$^{*\triangle}$	0.4630±0.0670$^{*\triangle}$
	1/8MIC	0.2937±0.0261$^{*\triangle}$	0.1722±0.0572$^{*\triangle}$	0.1772±0.0466$^{\triangle}$
	1/16MIC	0.1440±0.0120$^{*\triangle}$	0.1079±0.0542$^{\triangle}$	0.0759±0.0352$^{\triangle}$

Note: * denotes P<0.05 compared with blank control group, $^{\triangle}$ denotes P<0.05 compared with positive control group.

Table S3. Effects of tea polyphenols and theaflavins on biofilm biomass of cariogenic bacteria.

		OD value		
		S.m	*S.s*	*A.v*
Positive control group		0.134±0.024	0.155±0.038	0.173±0.031
Blank control group		0.751±0.047	0.825±0.054	0.876±0.046
Tea polyphenols	1/2MIC	0.288±0.023$^{*\triangle}$	0.308±0.026*	0.318±0.039*
	1/4MIC	0.356±0.026$^{*\triangle}$	0.396±0.037*	0.398±0.043$^{*\triangle}$
	1/8MIC	0.567±0.048$^{\triangle}$	0.627±0.034$^{\triangle}$	0.686±0.050$^{\triangle}$
	1/16MIC	0.684±0.036$^{\triangle}$	0.746±0.055$^{\triangle}$	0.863±0.014$^{\triangle}$
Theaflavins	1/2MIC	0.231±0.030*	0.246±0.048*	0.289±0.048*
	1/4MIC	0.404±0.010$^{*\triangle}$	0.381±0.039*	0.489±0.036$^{*\triangle}$
	1/8MIC	0.543±0.059$^{\triangle}$	0.608±0.033$^{\triangle}$	0.704±0.066$^{\triangle}$
	1/16MIC	0.692±0.031$^{\triangle}$	0.744±0.012$^{\triangle}$	0.843±0.052$^{\triangle}$

Note: * denotes P<0.05 compared with blank control group, $\triangle$ denotes P<0.05 compared with positive control group.

Table S4: Effects of tea polyphenols and theaflavins on the percentage of viable bacteria in the cariogenic biofilm.

		Percentage of viable bacteria		
		S.m	*S.s*	*A.v*
Positive control group		0.2912±0.0789	0.3074±0.0979	0.2323±0.0951
Blank control group		0.8890±0.0224	0.9564±0.0107	0.9743±0.0124
Tea polyphenols	1/2MIC	0.4611±0.0483*	0.3993±0.0718*	0.4667±0.0778*
	1/4MIC	0.5898±0.0337*	0.5836±0.0475*	0.6180±0.0491*
	1/8MIC	0.6525±0.0723	0.6552±0.0372*	0.7187±0.0523$^{\triangle}$
	1/16MIC	0.7300±0.0848$^{\triangle}$	0.7520±0.0537	0.7970±0.0639$^{\triangle}$
Theaflavins	1/2MIC	0.4128±0.0634*	0.4033±0.0799*	0.4383±0.0842*
	1/4MIC	0.5403±0.0910	0.5522±0.0794	0.5960±0.1170
	1/8MIC	0.6978±0.0653$^{\triangle}$	0.7168±0.0524	0.7563±0.0546$^{\triangle}$
	1/16MIC	0.7988±0.0541$^{\triangle}$	0.8368±0.0862$^{\triangle}$	0.8890±0.0290$^{\triangle}$

Note: * denotes P<0.05 compared with blank control group, $\triangle$ denotes P<0.05 compared with positive control group.
